# Atezolizumab/bevacizumab or lenvatinib in hepatocellular carcinoma: Multicenter real-world study with focus on bleeding and thromboembolic events

**DOI:** 10.1016/j.jhepr.2024.101065

**Published:** 2024-04-08

**Authors:** Najib Ben Khaled, Marie Möller, Leonie S. Jochheim, Catherine Leyh, Ursula Ehmer, Katrin Böttcher, Matthias Pinter, Lorenz Balcar, Bernhard Scheiner, Alexander Weich, Hans Benno Leicht, Valentina Zarka, Liangtao Ye, Julia Schneider, Ignazio Piseddu, Osman Öcal, Monika Rau, Friedrich Sinner, Marino Venerito, Simon Johannes Gairing, Friedrich Förster, Julia Mayerle, Enrico N. De Toni, Andreas Geier, Florian P. Reiter

**Affiliations:** 1 Department of Medicine II, University Hospital, LMU Munich, Munich, Germany; 2 Division of Hepatology, Department of Medicine II, University Hospital Würzburg, Würzburg, Germany; 3 Department of Gastroenterology and Hepatology, University Hospital Essen, University of Duisburg-Essen, Essen, Germany; 4 Department of Internal Medicine II, University Hospital Rechts der Isar, TUM School of Medicine and Health, Department Clinical Medicine, Munich, Germany; 5 Division of Gastroenterology and Hepatology, Department of Internal Medicine III, Medical University of Vienna, Vienna, Austria; 6 Division of Gastroenterology, Department of Medicine II, University Hospital Würzburg, Würzburg, Germany; 7 Digestive Diseases Center, The Seventh Affiliated Hospital, Sun Yat-sen University, Shenzhen, China; 8 Department Radiology, University Hospital, LMU Munich, Munich, Germany; 9 Department of Gastroenterology, Hepatology and Infectious Diseases, Otto-von-Guericke University Hospital Magdeburg, Magdeburg, Germany; 10 Department of Medicine I, University Medical Center of the Johannes-Gutenberg University Mainz, Germany

**Keywords:** Hepatocellular carcinoma, Immunotherapy, Tyrosine kinase inhibition

## Abstract

**Background & Aims:**

Atezolizumab/bevacizumab (atezo/bev) and lenvatinib have demonstrated efficacy as first-line therapies for hepatocellular carcinoma (HCC). However, vascular endothelial growth factor (VEGF) inhibition with these therapies may be associated with the risk of bleeding and thromboembolic events. In this study, we evaluated the efficacy and safety with focus on the bleeding and thromboembolic events of atezo/bev *vs*. lenvatinib in a large, multicenter real-world population.

**Methods:**

This study is based on HCC cohorts from seven centers in Germany and Austria. Incidences of bleeding or thromboembolic events and efficacy outcomes were assessed and compared.

**Results:**

In total, 464 patients treated with atezo/bev (n = 325) or lenvatinib (n = 139) were analyzed. Both groups were balanced with respect to demographics, presence of liver cirrhosis, and variceal status. Duration of therapy did not differ between groups. Within 3 months of therapy, bleeding episodes were described in 57 (18%) patients receiving atezo/bev compared with 15 (11%) patients receiving lenvatinib (*p* = 0.07). Variceal hemorrhage occurred in 11 (3%) patients treated with atezo/bev compared with 4 (3%) patients treated with lenvatinib (*p* = 0.99). Thromboembolic events were reported in 19 (6%) of patients in the atezo/bev cohort compared with 5 (4%) patients in the lenvatinib cohort (*p* = 0.37). In addition, incidence of overall bleeding, variceal hemorrhage, and thromboembolic events did not differ significantly in patients who received either atezo/bev or lenvantinib for 6 months.

**Conclusions:**

Safety considerations related to bleeding and thromboembolic events may not be helpful in guiding clinical decision-making when choosing between atezo/bev and lenvatinib.

**Impact and implications:**

The inhibition of VEGF by current first-line therapies for HCC, such as atezolizumab/bevacizumab or lenvatinib, may be associated with the risk of bleeding and thromboembolic events. Studies comparing the incidence of these side effects between atezolizumab/bevacizumab and lenvatinib, which are preferred treatments over sorafenib for HCC, are needed. Differences in this side effect profile may influence the choice of first-line therapy by treating physicians. Because no significant differences were observed regarding bleeding or thromboembolic events between both therapies in the present study, we conclude that safety considerations related to these events may not be helpful in guiding clinical decision-making when choosing between atezolizumab/bevacizumab and lenvatinib.

## Highlights

• Evaluation of the efficacy and safety of atezolizumab/bevacizumab and lenvatinib in a large real-world population of 464 patients with HCC.
• Compared with lenvatinib, atezolizumab/bevacizumab improves ORR.
• No significant differences in bleeding or thromboembolic events were observed.
• In atezolizumab/bevacizumab-treated patients, spleen size and history of variceal bleeding were associated with GI-bleeding risk, while anticoagulation was associated with non-GI bleeding risk.

## Graphical abstract

**Figure:**
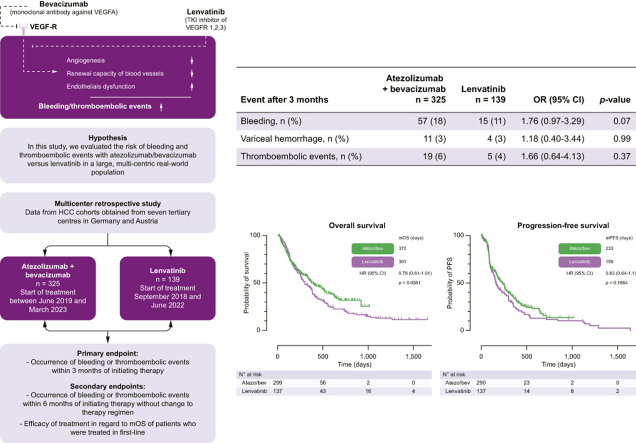

## Introduction

Hepatocellular carcinoma (HCC) ranks among the five leading causes of cancer-related death worldwide.^1^ Most patients are diagnosed at or progress to an advanced stage, necessitating systemic therapy.^2^^,^^3^ Fortunately, the field of systemic therapy for HCC has rapidly evolved in the last few years with the emergence of several new, effective regimens for its treatment.[4], [5], [6] Currently, four therapies have gained approval for use in first-line treatment, namely, sorafenib,^7^^,^^8^ lenvatinib,^9^ atezolizumab/bevacizumab (atezo/bev),^10^ and, most recently, durvalumab with or without tremelimumab.^11^ All these mentioned therapies were investigated in comparison with sorafenib. Comprehensive evaluations of efficacy and safety between the other systemic options are widely lacking.[9], [10], [11]

The pivotal IMbrave150 trial, which investigated the use of atezo/bev compared with sorafenib in patients with unresectable HCC, reported the superiority of atezo/bev with respect to median overall survival (OS), progression-free survival (PFS), and objective response rates (ORRs).^10^ Furthermore, the tyrosine kinase inhibitor lenvatinib demonstrated non-inferiority with respect to survival and higher PFS and ORR compared with sorafenib in a large phase III trial, whereas superiority in survival was not demonstrated for lenvatinib.^9^ These compelling findings have led to both regimens becoming preferred first-line alternatives to sorafenib for the treatment of HCC.^5^ Despite the promising results seen with atezo/bev and lenvatinib, concerns persist regarding the administration of anti-vascular endothelial growth factor (VEGF) inhibitors, especially bevacizumab, in patients with underlying chronic liver disease owing to the risk of bleeding.^12^ These perceptions are fueled by the strictly selected patients included in the IMbrave150 and REFLECT trials.^9^^,^^10^ The study populations may not represent patients with significant portal hypertension, who are frequently treated in real-world situations.

At the center of these concerns is VEGF, a key mediator of angiogenesis responsible for orchestrating the renewal of blood vessels in response to trauma.^13^ Impairment or inhibition of VEGF signaling compromises the repair of blood vessels, potentially leading to bleeding or thromboembolic events as a result of the exposure of subendothelial collagen.^14^ The side effects of anti-VEGF therapies are of particular concern in patients with HCC. Owing to the frequent coexistence of underlying chronic liver disease, these patients are *per se* at a higher risk for bleeding^15^ and/or thromboembolic events.^16^ Earlier studies that investigated bevacizumab before the era of immune checkpoint inhibitors (ICIs) for the treatment of HCC showed a high risk for variceal bleeding and reported rates of variceal hemorrhage of up to 10% in phase II trials.[17], [18], [19] Intriguingly, the IMbrave 150 trial reported a considerably lower incidence of variceal hemorrhage in only 2.4% of patients treated with atezo/bev compared with 0.6% of patients treated with sorafenib.^10^ In addition to this numerically higher occurrence of variceal bleeding events in the atezo/bev arm, the Imbrave150 study also reported higher rates of overall bleeding (25.2 *vs*. 17.3%) and arterial thromboembolic events (2.7 *vs*. 1.3%) in patients treated with atezo/bev compared with sorafenib. Given lenvatinib’s superiority in PFS and ORR over sorafenib, it represents a frequently preferred alternative agent in the first-line treatment. Lenvatinib, however, is a stronger VEGF inhibitor than sorafenib,[20], [21], [22] which may compromise its safety when compared with atezo/bev in clinical practice.

At present, only a limited body of evidence exists for comparing these two first-line therapies.[23], [24], [25] Most published studies have predominantly emphasized efficacy rather than the profiles of side effects. Moreover, evaluations of side effects in many studies have been conducted from an intention-to-treat perspective, lacking analyses to determine if bleeding rates were associated with follow-up therapies. Given these gaps, a thorough examination, particularly in a real-world population that typically does not reflect a selectively chosen study group, holds significant scientific importance to contribute evidence on this topic.

As a result, a comprehensive and detailed investigation into the safety and efficacy of atezo/bev *vs*. lenvatinib in a real-world setting becomes imperative, forming the rationale for the present study. The intention is to analyze parameters of efficacy and safety within a large, real-world population.

## Patients and methods

### Patient population

This study was initiated by the IMMUreal study group. The objective of the IMMUreal study group is to investigate the efficacy of immunotherapeutic agents for the treatment of liver tumors. Patients were recruited from six centers in Germany (University Hospital LMU Munich, University Hospital Essen, Klinikum Rechts der Isar TU Munich, University Hospital Magdeburg, University Medical Center Mainz, and University Hospital Würzburg) and one center in Austria (Medical University of Vienna). All patients included in our study had a confirmed diagnosis of HCC based on histopathological findings or typical diagnostic imaging, following the EASL criteria.^26^^,^^27^ This study was approved by local authorities (Ethikkommission an der Julius-Maximilians-Universität Würzburg, 156/21-me) and conducted in accordance with the Declaration of Helsinki. We used the STROBE cohort checklist and followed the European Society for Medical Oncology (ESMO) Guidance for Reporting Oncology real-World evidence (GROW) when writing our report.^28^^,^^29^

### Treatments

Patients received the following treatment regimens: (1) atezo plus bev, where atezo was administered i.v. at a dose of 1200 mg and bev at 15 mg per kg of body weight every 3 weeks, and (2) lenvatinib at 12 mg orally once daily for patients with body weight ≥60 kg and at 8 mg once daily for patients <60 kg. The following procedures were conducted during the treatment phase: Patients were monitored through clinical, laboratory, and imaging assessments according to the standard of care, following the German HCC guidelines.^30^ During the visits, patients’ vital signs were measured, and laboratory tests were performed including a complete blood count, serum chemistry, parameters of liver function, and alpha-fetoprotein levels. If symptoms occurred, additional tests such as a focused physical examination, further laboratory tests, ECG, or imaging were performed at the discretion of the local investigator. An adverse events assessment was performed at each visit. Tumor response was assessed every 8 to 12 weeks using computed tomography and/or magnetic resonance imaging.

### Endpoints

The primary question of this study was to investigate the occurrence of bleeding or thromboembolic events within 3 months of therapy initiation. Secondary safety questions included the rates of bleeding or thromboembolic events within 6 months of therapy initiation without change to the therapeutic regimen. Data on safety with a focus on bleeding and thromboembolic events were collected. Secondary efficacy endpoints included OS and response rates in patients treated in the first line. OS was defined as the time from treatment initiation to death from any cause. Patients without an OS event or patients who were lost to follow-up were censored on their last contact day. Treatment response was analyzed using routine computed tomography or magnetic resonance imaging until death or the end of treatment. Radiological response was categorized as complete response (CR), partial response (PR), stable disease (SD), or progressive disease (PD) by the local investigator and/or radiologist in accordance with either Response Evaluation Criteria in Solid Tumors (RECIST) version 1.1 or modified RECIST (mRECIST). Therefore, the study objectives involve a descriptive evaluation of two real-world cohorts treated with either atezo/bev or lenvatinib. Furthermore, the comparison between safety and efficacy data is classified as an analytical objective of the present study.

### Statistical analysis

The statistical analysis of this study used two datasets. Patients who received atezo/bev or lenvatinib beyond the first line were included in the analysis for safety, whereas those only in the first line were included for the analysis of efficacy (Table 1). The efficacy dataset focused on patients, who received atezo/bev or lenvatinib in the first line in the approved indications.^31^ Statistical calculations were performed using GraphPad Prism 9 (GraphPad Software, San Diego, CA, USA). Baseline characteristics were summarized using descriptive statistics. The normal distribution of variables was assessed through the Shapiro–Wilk test and inspection of qq-plots. Continuous variables were presented as mean plus standard deviation and compared using either the *t* test or the Mann–Whitney *U* test, depending on their distribution. Categorical variables were reported as numbers and percentages. Fisher’s exact test was used for comparing categorical variables. Kaplan–Meier analysis was performed to calculate the median OS, which was compared using the log-rank test. Hazard ratios for events were estimated through univariate and multivariate logistic regression. Receiver operating characteristic (ROC) curve analysis was used to determine the optimal cut-off values for spleen size that could produce the highest sensitivity and specificity in predicting bleeding events. Values of *p* less than 0.05 were considered statistically significant.

**Table 1:** Baseline characteristics.

Patient characteristics	Atezolizumab + bevacizumab (n = 325)	Lenvatinib (n = 139)
Age (years), median (IQR)	69 (25–96)	68 (31–85)
Sex female, n (%)	75 (23)	25 (18)
Liver cirrhosis, n (%)	233 (72)	103 (74)
Child–Pugh A	175 (75)	85 (83)
Child–Pugh B	40 (17)	16 (16)
Child–Pugh C	13 (6)	2 (2)
Unknown	5 (2)	0 (0)
Gastroesophageal varices, n (%)	134 (41)	50 (36)
Esophageal I°	85 (63)	28 (56)
Esophageal II°	40 (30)	10 (20)
Esophageal III°	3 (2)	7 (14)
Gastric or fundic	4 (3)	5 (10)
Others (rectal or downhill varices)	2 (1)	0 (0)
Non-selective beta-blockers	67 (21)	10 (7)
Banding	21 (6)	13 (9)
Non-selective beta-blockers + banding	21 (6)	8 (6)
No EGD available	22 (7)	16 (12)
History of variceal hemorrhage, n (%)	19 (6)	8 (6)
Portal vein thrombosis∗, n (%)	97 (30)	37 (27)
Anticoagulants, n (%)	95 (29)	38 (27)
LMWH	60 (18)	23 (17)
VKA	3 (1)	0 (0)
DOAC	30 (9)	15 (11)
Unknown	2 (1)	0 (0)
Antiplatelet agents, n (%)	83 (26)	31 (22)
BCLC stage, n (%)		
BCLC A	7 (2)	1 (<1)
BCLC B	82 (25)	30 (22)
BCLC C	224 (69)	103 (74)
BCLC D	13 (4)	5 (4)
Line of systemic therapy, n (%)		
First line	311 (96)	137 (99)
Second line	10 (3)	1 (<1)
Third line	2 (<1)	1 (<1)
Unknown	2 (<1)	0 (0)
Prior non-systemic therapy		
Patients/procedures, n/n	43/56	37/56
Resection, n (procedures, %)	12 (21)	11 (20)
MWA/RFA, n (procedures, %)	7 (13)	6 (11)
TACE, n (procedures, %)	27 (48)	17 (30)
SIRT, n (procedures, %)	1 (2)	20 (36)
SBRT, n (procedures, %)	9 (16)	2 (4)
Etiology of underlying liver disease, n (%)		
HBV/HCV	86 (26)	40 (29)
Non-viral	222 (68)	87 (63)
Unknown	17 (5)	12 (9)
Extrahepatic spread∗, n (%)	103 (44)	45 (55)
Macrovascular invasion∗, n (%)	83 (35)	22 (27)

∗ Data regarding the extrahepatic spread and macrovascular invasion were available for 236 patients treated with atezo/bev and 82 patients treated with lenvatinib. BCLC, Barcelona Clinic Liver Cancer; DOAC, direct oral anticoagulants; EGD, esophagogastroduodenoscopy; LMWH, low-molecular-weight heparins; MWA, microwave ablation; RFA, radiofrequency ablation; SBRT, stereotactic body radiation therapy; SIRT, selective internal radiation therapy; TACE, transarterial chemoembolization; VKA, vitamin K antagonists.

### Study design

In this retrospective multicenter study, source data from several prospective HCC cohorts were analyzed. Given the unlikelihood of a prospective head-to-head trial of atezo/bev *vs*. lenvatinib being performed or supported by industry partners, an analysis of patient cohorts is deemed the most valid approach to address these important questions regarding differences in efficacy and safety between first-line therapies. Furthermore, this analysis has the advantage of including patients treated in real-world situations – a population often not accurately represented in oncological studies in the HCC field, where usually only patients with very good liver function are enrolled.

### Exclusion criteria

The selection of therapy was based on investigator choice. Given that the primary objective of this study was to present a pure real-world situation, we did not exclude any patients with HCC from the study, except for those who refused to provide informed consent. Patients with mixed HCC/CCC tumors or fibrolamellar HCCs were not recruited for the present study.

### Data source and study data management

Data were obtained from medical records, patients’ reports, or both during the recruitment process for observational prospective patient cohorts at each center.

Source data were provided by each center in an anonymized manner through a pre-specified form. FPR facilitated the merging of all the data for further analysis. Throughout the analysis, no duplicated cases were identified. The absence of duplicates is largely attributed to the diverse geographical locations.

The datasets were collected in May 2023. Subsequently, completion of data was verified by FPR and NBK, and quality controls and validation were performed thereafter.

## Results

### Baseline characteristics

**Fig. 1:**
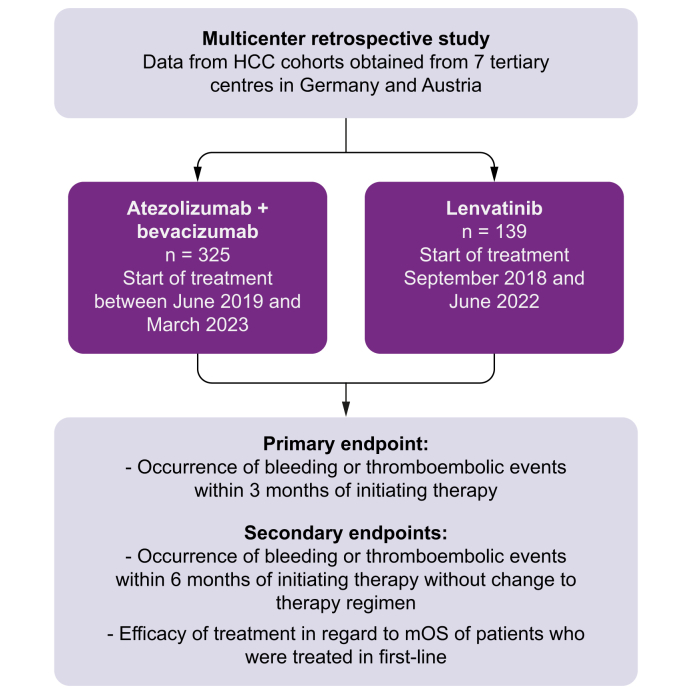
Study flowchart. HCC, hepatocellular carcinoma; mOS, median overall survival.

**Fig. 2:**
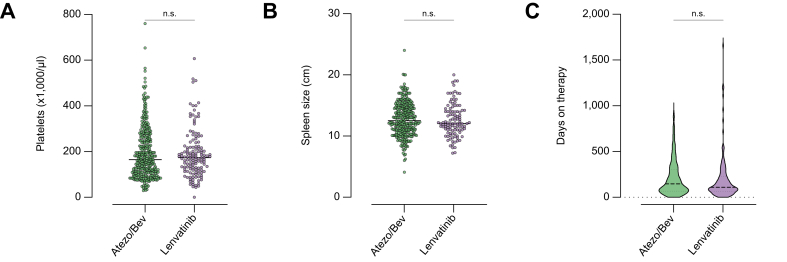
Baseline characteristics. (A) Platelets and (B) spleen size as surrogates of portal hypertension were not different between the groups (platelets: *p* = 0.82; spleen size: *p* = 0.51). (C) Time on therapy was reported as 214.6 ± 193.9 days in the atezo/bev group and 195.9 ± 248.4 days in the lenvatinib group, which was not significantly different (*p* = 0.13). For comparison of both groups, the normal distribution of variables was assessed using the Shapiro–Wilk test and through inspection of qq-plots. Continuous variables were compared using either the *t* test or the Mann–Whitney *U* test, depending on their distribution. atezo/bev, atezolizumab/bevacizumab.

Data from 464 patients who were treated between September 2018 and March 2023 with either atezo/bev (n = 325) or lenvatinib (n = 139) were analyzed in this study (Fig. 1). The baseline characteristics are illustrated in Table 1. Underlying liver cirrhosis was present in most cases, with 233 (72%) patients in the atezo/bev group and 103 (74%) patients in the lenvatinib group (*p* = 0.65). The overall prevalence of gastroesophageal varices was balanced between both groups (atezo/bev n = 134 [41%] *vs*. lenvatinib n = 50 [36%], *p* = 0.30). In the atezo/bev group, there were statistically significantly fewer patients with esophageal varices of grade III (atezo/bev n = 3 [1%] *vs*. lenvatinib n = 7 [5%], *p* = 0.01). The percentage of prophylactic therapy with non-selective beta-blocker (NSBB) therapy, variceal banding, or both was statistically significantly higher in the atezo/bev group (n = 109 [34%]) than in the lenvatinib group (n = 31 [22%]) (*p* = 0.02). Other factors that predispose to bleeding or thromboembolic events did not differ between both groups, such as the use of anticoagulation (atezo/bev n = 95 [29%] *vs*. lenvatinib n = 38 [27%], *p* = 0.74), antiplatelet therapy (atezo/bev n = 83 [26%] *vs*. lenvatinib n = 31 [22%], *p* = 0.48), or history of variceal bleeding (atezo/bev n = 19 [6%] *vs*. lenvatinib n = 8 [6%], *p* >0.99). There was no statistically significant difference in patients who did not undergo index endoscopy (atezo/bev n = 22 [7%] *vs*. lenvatinib n = 16 [12%], *p* = 0.10). Surrogate parameters for portal hypertension such as spleen size (atezo/bev 12.7 ± 2.8 *vs*. lenvatinib 12.6 ± 2.7, *p* = 0.51) or platelet count (atezo/bev 194 ± 115 *vs*. lenvatinib 191 ± 105, *p* = 0.82) did not differ between both groups (Fig. 2A and B). The duration of therapy was reported as 214.6 ± 193.9 days in the atezo/bev group and 195.9 ± 248.4 days in the lenvatinib group (*p* = 0.13) (Fig. 2C). Both groups were balanced with respect to underlying etiology and Barcelona Clinic Liver Cancer (BCLC) stage (Table 1). Most patients received atezo/bev (n = 311 [96%]) or lenvatinib (n = 137 [99%]) as first-line systemic therapy (*p* = 0.17). Data on the evidence of macrovascular invasion and extrahepatic spread were available in 236 (73%) patients treated with atezo/bev and in 82 (59%) patients treated with lenvatinib. In this regard, 103 (44%) patients treated with atezo/bev *vs*. 45 (55%) patients treated with lenvatinib exhibited extrahepatic spread (*p* = 0.09), whereas 83 (35%) and 22 (27%) patients respectively showed macrovascular invasion (*p* = 0.18). Of the 325 patients treated with atezo/bev, 303 (93%) started immediately with atezo/bev as a combination regimen, whereas 21 (6%) started with atezo monotherapy. Of these 21 patients, 7 (2%) received bev at the second cycle, and 5 (2%) over the following 1 to 6 months. In one patient, it was unclear whether atezo was started with bev. Of the 21 patients who started with atezo monotherapy, 9 (43%) never received bev in the course of treatment.

### Incidence of bleeding, variceal hemorrhage, and thromboembolic events

As the primary objective, we evaluated the evidence of bleeding or thromboembolic events in the safety analysis dataset. In the atezo/bev cohort, the median time to bleeding was 2.9 months (IQR 1.1–6.9 months), and the median time to a thromboembolic event was 3.0 months (IQR 2.3–5.8 months). In patients treated with lenvatinib, the median time to bleeding was 3.4 months (IQR 2.8–7.0 months), and the median time to a thromboembolic event was 3.5 months (IQR 2.3–7.3 months). After 3 months of treatment, there was no significant difference in overall bleeding episodes between both groups (atezo/bev n = 57 [18%] *vs*. lenvatinib n = 15 [11%]; odds ratio [OR] 1.76, 95% CI 0.97–3.29; *p* = 0.07) (Table 2). The occurrence of variceal hemorrhage in the first 3 months of therapy was low in both groups, approximately 3% (atezo/bev n = 11 [3%] *vs*. lenvatinib n = 4 [3%]; OR 1.18, 95% CI 0.40–3.44; *p* = 0.99) (Table 2). Thromboembolic events did not differ significantly between both groups (atezo/bev n = 19 [6%] vs. lenvatinib n = 5 [4%]; OR 1.66, 95% CI 0.63–4.13; *p* = 0.37) (Table 2). These results were reproduced after excluding patients who died in the first 3 months (Table S2).

**Table 2:** Bleeding and thromboembolic events after 3 months of therapy.

Event	Atezolizumab + bevacizumab (n = 325), n (%)	Lenvatinib (n = 139), n (%)	OR (95% CI)	*p* value
Bleeding	57 (18)	15 (11)	1.76 (0.97–3.29)	0.07
Variceal hemorrhage	11 (3)	4 (3)	1.18 (0.40–3.44)	0.99
Thromboembolic events	19 (6)	5 (4)	1.66 (0.64–4.13)	0.37

Categorical variables are reported as numbers and percentages. Fisher's exact test was used for comparing categorical variables. OR, odds ratio.

Because the median time to onset of bleeding is approximately 12 weeks with both agents,^9^ we also evaluated the bleeding and thromboembolic events within 6 months of therapy without alterations to the treatment regimen (Table 3). At 6 months, 111 (34%) and 31 (22%) patients of the overall cohort were still on therapy with either atezo/bev or lenvatinib, respectively. In these subgroups, we did not find statistical differences with respect to overall bleeding events (atezo/bev n = 32 [29%] *vs*. lenvatinib n = 6 [19%]; OR 1.69, 95% CI 0.65–4.41; *p* = 0.36) or variceal hemorrhage (atezo/bev n = 10 [9%] *vs*. lenvatinib n = 2 [6%]; OR 1.44, 95% CI 0.35–6.84; *p* >0.99) between both groups. Thromboembolic events did not differ between both groups after 6 months of therapy (atezo/bev n = 13 [13%] *vs*. lenvatinib n = 4 [11%]; OR 1.17, 95% CI 0.39–3.47; *p* >0.99). In a further analysis, we evaluated all bleeding episodes until 6 months of therapy, for which a grading according to CTCAE classification was available. For this analysis, patients who underwent a switch of therapy after 3 months were included. In 70 (22%) cases of patients who received systemic therapy with atezo/bev, as well as in 18 (13%) of those treated with lenvatinib, bleeding episodes with reported CTCAE classification were available for up to 6 months. Here, we did not find differences concerning high-grade bleeding (≥III°), which were reported in 46 (66%) patients in the atezo/bev group and 11 (61%) patients in the lenvatinib group (*p* = 0.78).

**Table 3:** Bleeding and thromboembolic events in patients who received therapy for more than 6 months.

Event	Atezolizumab + bevacizumab (n = 111), n (%)	Lenvatinib (n = 31), n (%)	OR (95% CI)	*p* value
Bleeding	32 (29)	6 (19)	1.69 (0.65–4.41)	0.36
Variceal hemorrhage	10 (9)	2 (6)	1.44 (0.35–6.84)	0.99
Thromboembolic events	13 (13)	4 (11)	1.17 (0.39–3.47)	0.99

Fisher's exact test was used for comparing categorical variables. OR, odds ratio.

### Risk factors for bleeding in atezo/bev-treated patients

In the next step, we aimed to identify risk factors for gastrointestinal (GI), variceal, and non-GI bleeding in patients treated with atezo/bev in the first line. Spleen size (OR 1.2, 95% CI 1.0–1.3; *p* = 0.007) and history of variceal bleeding (OR 3.9, 95% CI 1.4–10; *p* = 0.007) were significantly associated with the risk for GI bleeding in univariate regression analysis (Table 4). Both parameters persisted as risk factors for GI bleeding in multivariate regression analysis. Concerning variceal bleeding, we could identify spleen size (OR 1.2, 95% CI 1.0–1.4; *p* = 0.04) and the presence of high-grade (II–III) esophageal varices (OR 8.6, 95% CI 2.2– 42; *p* = 0.003) as significant risk factors in univariate analysis (Table 4). Because of the low event rate of variceal bleeding, no multivariate analysis was conducted. Anticoagulation was significantly associated with an increased risk for non-GI bleeding (OR 2.3, 95% CI 1.1–4.9; *p* = 0.02) (Table 4), but not with GI or variceal bleeding. In particular, the use of direct oral anticoagulants (DOAC) was linked to the occurrence of non-GI bleeding episodes in our cohort. Specifically, 25.8% of patients on DOAC developed non-GI bleeding while being treated with atezo/bev (n = 8/31), compared with 10% of patients on low-molecular-weight heparin (n = 6/60) and 7.8% of patients without anticoagulation (n = 18/231). Only three patients in the cohort received a vitamin K antagonist, and no bleeding events occurred in these patients. We did not identify significant associations between the risk of GI, variceal, and non-GI bleeding and age, sex, Child–Pugh B/C score, platelet counts, or use of antiplatelet drugs such as aspirin. The presence of liver cirrhosis was not associated with an increased risk of GI and non-GI bleeding. For variceal bleeding, a regression model with cirrhosis as a variable was not constructed, as variceal bleeding occurred only in patients with liver cirrhosis. Because spleen size was found to be significantly associated with the risk of GI and variceal bleeding, critical spleen size thresholds were analyzed using ROC curves. The AUC of spleen size was 0.861 in predicting variceal bleeding, with a cut-off size of 13.36 cm, and 0.799 in predicting GI bleeding, with a cut-off size of 12.9 cm (Fig. S1). At the optimal cut-off value, the sensitivity and specificity for spleen size were 78.9 and 61.3% in predicting variceal bleeding and 71.4 and 55.9% in predicting GI bleeding, respectively. Finally, as HCC in patients with liver cirrhosis occurred in approximately 70% of all patients in this cohort (Table 1) and risk factors for bleeding might differ among patients with and without cirrhosis, we repeated the analysis including only patients with cirrhosis. In this subgroup analysis, the previously identified risk factors could be confirmed. The risk for GI bleeding was significantly associated with previous bleeding, whereas the risk for variceal bleeding was associated with the presence of high-grade varices at baseline gastroscopy (Table S1). For non-GI bleeding, anticoagulation persisted as a risk factor in patients with cirrhosis.

**Table 4:** Potential risk factors for gastrointestinal bleeding, variceal bleeding, and non-gastrointestinal bleeding in patients treated with atezo/bev.

Variable	Univariate analysis			Multivariate analysis		
	OR	95% (CI)	*p* value	OR	95% (CI)	*p* value
**Gastrointestinal bleeding**						
Age	0.98	0.95–1.0	0.15			
Sex, male	1.1	0.55–2.5	0.75			
Liver cirrhosis	2.1	0.99–5.0	0.07			
CPS B (CPS A as reference)	1.3	0.52–3.0	0.54			
CPS C (CPS A as reference)	1.6	0.34–5.5	0.51			
MVI	0.96	0.44–2.0	0.91			
Platelets	1	0.99–1.0	0.14			
**Spleen size**	**1.2**	**1.0–1.3**	**0.007**	**1.1**	**1.0–1.3**	**0.03**
Presence of varices	1.8	0.91–3.5	0.09			
**History of variceal bleeding**	**3.9**	**1.4–10**	**0.007**	**3.0**	**1.0–8.4**	**0.04**
NSBB	0.8	0.37–1.6	0.54			
Prior EBL	2.1	0.91–4.5	0.07			
Anticoagulation	1.5	0.75–2.8	0.26			
Antiplatelets	0.76	0.34–1.6	0.47			
**Variceal bleeding**						
Age	0.96	0.92–1.0	0.07			
Sex, male	2.2	0.59–14	0.31			
CPS B (CPS A as reference)	2.4	0.70–7.1	0.14			
CPS C (CPS A as reference)	1.4	0.072–8.1	0.77			
MVI	1.6	0.51–5.1	0.40			
Platelets	1	0.99–1.0	0.18			
**Spleen size**	**1.2**	**1.0–1.4**	**0.04**			
**Presence of varices**	**4.5**	**1.4–21**	**0.02**			
Esophageal varices grade I	2.7	0.59–14	0.2			
**Esophageal varices grades II–III**	**8.6**	**2.2–42**	**0.003**			
History of variceal bleeding	2.5	0.37–9.8	0.26			
NSBB	1.2	0.38–3.5	0.7			
Prior EBL	2.4	0.64–7.2	0.15			
Anticoagulation	2	0.68–5.4	0.2			
**Non-gastrointestinal bleeding**						
Age	0.99	0.95–1.0	0.53	1	0.97–1.0	0.74
Sex, male	2.2	0.84–7.7	0.14	2.1	0.79–7.4	0.17
Liver cirrhosis	2.3	0.92–6.9	0.10			
CPS B/C (CPS A as reference)	0.24	0.037–0.83	0.05 ns			
Platelets	1	1.0–1.0	0.57			
**Anticoagulation**	**2.3**	**1.1–4.9**	**0.02**	**2.2**	**1.0–4.7**	**0.04**
Antiplatelets	1.5	0.65–4.3	0.36			

ORs for the risk of bleeding were calculated using both univariate and multivariate logistic regression models. Liver cirrhosis was not included in the model for risk factors of variceal bleeding, because the presence of liver cirrhosis and the occurrence of variceal bleeding were linearly dependent predictors causing estimation instability unsuitable for logistic regression analysis. The bold emphasis indicate statistical significant results. azeto/bev, atezolizumab/bevacizumab; CPS, Child–Pugh score; EBL, endoscopic band ligation; MVI, macrovascular invasion; NSBB, non-selective beta-blocker; OR, odds ratio.

### Variceal hemorrhage occurring in patients treated with atezo/bev

In a further subgroup analysis, we characterized all patients who experienced an episode of variceal hemorrhage under or after therapy with atezo/bev. We identified 18 patients who developed variceal bleeding (Table 5). Most of these patients presented with good liver function (Child–Pugh A, n = 12 [67%]) (Table 5). Of the 18 patients, 11 (61%) had gastroesophageal varices at baseline. Most patients exhibited low-grade varices before the start of therapy (no varices, n = 3 [17%]; I°, n = 4 [22%]; II°, n = 6 [33%]; gastric or fundic varices, n = 1 [6%]) (Table 5). Moreover, 9 (50%) patients did not receive prophylactic treatment. History of variceal hemorrhage was recorded in 2 (11%) patients. Macrovascular invasion or portal vein thrombosis was described in 10 (56%) of the 18 patients. In addition, 7 (39%) patients who developed variceal bleeding were on anticoagulation, and none of the patients received antiplatelet therapy. The recorded rates of best response under atezo/bev showed PR in 7 (39%) patients, SD in 6 (33%) patients, and PD in 1 (6%) patient. In 3 (17%) patients, no response assessment was available.

**Table 5:** Summary of all episodes of variceal bleeding in patients receiving atezo/bev.

Characteristics and demographics	Value, n (%)
Liver cirrhosis	18 (100)
Child–Pugh A	12 (67)
Child–Pugh B	5 (28)
Child–Pugh C	1 (6)
Gastroesophageal varices	11 (61)
No varices	3 (17)
Esophageal I°	4 (22)
Esophageal II°	6 (33)
Esophageal III°	0 (0)
Gastric or fundic	1 (6)
Unknown status	3 (17)
No prophylaxis	9 (50)
Non-selective beta-blockers	4 (22)
Banding	4 (22)
Non-selective beta-blockers + banding	1 (6)
History of variceal hemorrhage	2 (11)
Macrovascular invasion/portal vein thrombosis	10 (56)
Anticoagulation	7 (39)
Best response	18 (100)
Complete response	0 (0)
Partial response	7 (39)
Stable disease	6 (33)
Progressive disease	1 (6)
Unknown	3 (17)

azeto/bev, atezolizumab/bevacizumab.

### Efficacy of atezo/bev *vs*. lenvatinib in first-line therapy

**Fig. 3:**
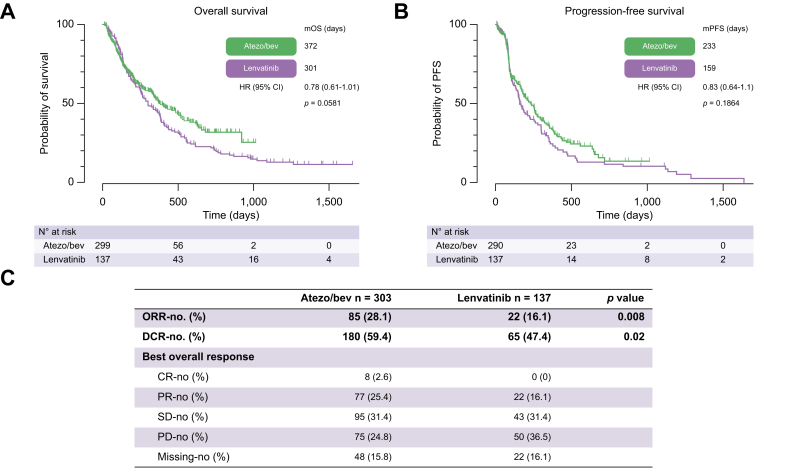
**Efficacy analysis of atezo/bev*****vs*****.lenvatinib in first-line therapy.** (A) mOS, (B) mPFS, and (C) best response according to local investigator evaluation were assessed in patients receiving either atezo/bev or lenvatinib in the first-line situation. Kaplan–Meier analysis was performed to calculate the median survival. Survival was compared using the log-rank test. Fisher’s exact test was used to compare response rates. atezo/bev, atezolizumab/bevacizumab; CR, complete response; DCR, disease control rate; HR, hazard ratio; mOS, median overall survival; mPFS, median progression-free survival; ORR, objective response rate; PD, progressive disease; PR, partial response; SD, stable disease.

Treatment outcomes were evaluated in the efficacy dataset consisting of patients who received atezo/bev or lenvatinib in the first line in the approved indications. In total, 303 patients treated with atezo/bev and 137 treated with lenvatinib were eligible for efficacy analysis. At a median follow-up of 19.3 months, 149 of 303 (49.2%) patients in the atezo/bev group had died, compared with 117 of 137 (85.4%) in the lenvatinib group. OS was 12.2 months with atezo/bev *vs*. 9.9 months with lenvatinib (hazard ratio [HR] 0.78, 95% CI 0.608–1.008; *p* = 0.058) (Fig. 3A). Patients treated with atezo/bev had a PFS of 7.7 months *vs*. 5.2 months with lenvatinib (HR 0.83, 95% CI 0.64–1.1; *p* = 0.19) (Fig. 3B). ORR and disease control rate (DCR) were significantly higher with atezo/bev (ORR, 28.1 *vs*. 16.1%, *p* = 0.008; DCR, 59.4 *vs*. 47.4%, *p* = 0.02) (Fig. 3C). Concerning viral or non-viral etiology, atezo/bev-treated patients with viral HCC had a longer OS than lenvatinib-treated patients with viral HCC (21.5 *vs*. 9.0 months; HR 0.61, 95% CI 0.369–0.995; *p* = 0.0477) (Fig. S2A). The OS in the non-viral groups was similar for atezo/bev *vs*. lenvatinib (HR 0.88, 95% CI 0.657–1.186; *p* = 0.4081) (Fig. S2B). At the time of data cut-off, 79 (30%) patients in the atezo/bev group and 72 (52%) patients in the lenvatinib group received more than one line of systemic treatment.

## Discussion

High rates of variceal bleeding were reported in previous studies performed in the ‘pre-ICI era’, when bevacizumab was investigated alone or in combination with either chemotherapies or erlotinib.^17^ The results from these studies raised concerns in the community regarding complications from portal hypertension in patients treated with atezo/bev.^12^

In recent years, several studies have compared the efficacy of atezo/bev and lenvatinib in a real-world setting.[23], [24], [25]^,^^32^ Some of these studies also reported adverse events related to bleeding. In this regard, a study conducted by Kim *et al.*^23^ in a cohort with a high incidence of underlying liver cirrhosis revealed GI bleeding rates of any grade as 2.7% in the lenvatinib group and 5.8% in the atezo/bev group. However, the rates of grade 3 or 4 GI bleeding events were comparable in both study groups (2.7% in lenvatinib *vs*. 3.5% in atezo/bev).^23^ The authors did not distinguish between variceal hemorrhage and general GI bleeding. Nevertheless, one might speculate that high-grade GI bleeding episodes are, in a relevant proportion, related to variceal bleeding. Assuming this, the results seem consistent with the data we report here. Another study published by Niizeki *et al.*^24^ reported significantly higher rates of bleeding events of any grade, as well as grades 3 and 4 bleeding events under atezo/bev compared with lenvatinib. These results need to be interpreted in the context of a significantly higher reported survival for atezo/bev than for lenvatinib in the same study,^24^ which increases the time available for the development of bleeding episodes. Furthermore, the reported bleeding events under lenvatinib, at only 0.6%, appear low when compared with a recent publication investigating bleeding episodes, with a specific focus on variceal bleeding under lenvatinib, from a large real-world cohort.^33^ This study reported variceal bleeding events of 3.61%, indicating that overall bleeding events extend beyond this range.

In the present study, we describe a rate of bleeding events in real-world patients receiving atezo/bev, with 3% showing variceal bleeding within 3 months of therapy. Furthermore, bleeding complications were not statistically significantly different from the first-line alternative with lenvatinib, which reflects a commonly preferred first-line therapy attributable to higher PFS and ORR compared with sorafenib.^9^

For an accurate interpretation of the non-different results regarding bleeding episodes between atezo/bev and lenvatinib reported here, it is important to emphasize a potential bias that could be related to an individual selection of therapy for patients at risk for bleeding. One might assume that patients with a high risk for bleeding were treated with lenvatinib rather than with atezo/bev, a fact that could influence the results of our study significantly. However, in this regard, we can state that the majority of patients treated with lenvatinib (123/139 [88%]) started therapy before October 27, 2020, the day atezo/bev was approved for treatment of HCC in Germany and Austria. This time frame suggests that bias resulting from individual selection might have minor relevance for the reported results here, as most patients (88%) received lenvatinib before the approval of atezo/bev. Potential reasons why we did not confirm the high rates of variceal bleeding events after 3 months of therapy compared with historical data may be related to greater attention paid to prophylactic management of portal hypertension in patients treated with atezo/bev, which emerged from these historical data.^17^ In this regard, we noticed that 110 of 134 patients (82%) in the atezo/bev group and 31 of 51 (62%) in the lenvatinib group who had varices received some kind of prophylactic therapy. Furthermore, we observed that 16 of 139 patients (12%) in the lenvatinib group had no recent esophagogastroduodenoscopy (EGD) available, whereas only 22 of 325 (7%) in the atezo/bev group received therapy with unknown variceal status. Although there was a numerical difference in the availability of index EGDs, we postulate that these rates (12 *vs*. 7%), which did not reach statistical significance, should not significantly affect the study results. However, the reported differences in prophylactic therapy might be relevant for the interpretation of the incidence of bleeding events observed. Despite a statistically significant difference in prophylactic therapy for variceal bleeding in both groups, we conclude that, in general, the bleeding rates after 3 months were not different between both groups. Furthermore, overall bleeding events, which should be more independent of prophylactic therapy for portal hypertension, did not differ significantly between both groups as well.

In our opinion, safety events need to be interpreted regarding survival and the possibility of a therapy crossover. The IMbrave150 trial demonstrated a significant increase in survival under atezo/bev compared with sorafenib.^10^ Thereby, the numerically higher rates of bleeding events reported there could also be influenced by the differences in survival, which simply provide patients with more time to experience bleeding events. Furthermore, a change of therapy in case of progression might bias the rates of bleeding as well. Therefore, we decided to focus on selecting the bleeding rates that occur within the first 3 months of therapy as the primary objective of this study. This choice aims to reduce the influence of a therapy crossover, which usually occurs after 3 months of therapy when the first staging scan is performed. Furthermore, this objective should not be significantly biased by differences in survival under therapy. In this regard, we observed an indifferent median duration of therapy in both groups, which exceeded 12 weeks (atezo/bev 209.9 ± 193.1 days *vs*. lenvatinib 195.9 ± 248.4 days, not significant). From these results, we conclude that neither a selection bias nor a bias resulting from crossover or different durations of therapy should be of relevance for the interpretation of the results reported here. However, the less intensive management of portal hypertension in the lenvatinib group might result in bleeding rates higher than those achievable with more consistent screening and treatment for portal hypertension.

In a subgroup of this study, we analyzed the data of all patients who developed variceal bleeding and had received therapy with atezo/bev. Interestingly, 12 of 18 patients had good liver function classified as Child–Pugh A. Although more patients in this subgroup had varices (61%) compared with the overall atezo/bev population (41%), the same percentage received prophylactic therapy (82%) in case of the presence of varices. Furthermore, the majority of patients (72%) experienced disease control, defined as PR or SD. From this pattern, it does not seem that the events of variceal bleeding were related to tumor progression, impaired liver function, or insufficient prophylaxis for portal hypertension. A potential explanation could stem from the observed disease control rates in this subgroup, as patients who respond to therapy have usually longer exposure to systemic therapy. This suggests that bleeding rates may be related to side effects from systemic tumor therapy. However, from the low number of events reported here (n = 18 patients with variceal bleeding) we can only speculate about this. In addition, it is important to underscore that the limited occurrence of variceal hemorrhage events constrains the establishment of a robust multivariate model.

We encourage the scientific community to pay attention in future studies to investigate whether the incidence of bleeding events is associated with the duration of therapy.

Furthermore, thromboembolic events were reported to be increased in patients treated with bevacizumab. In this regard, a meta-analysis conducted in patients receiving bevacizumab for treatment of colorectal cancer, analyzing 22 randomized controlled trials with a sample size of 13,185 patients, reported a relative risk for thromboembolism of 37%.^34^ In the present analysis, we did not find differences in thromboembolic events between atezo/bev and lenvatinib. It seems plausible that the VEGF inhibition, which is mediated by both agents, may result in equal risk rates. As most currently available systemic HCC therapies target VEGF, it will be an interesting question to explore whether there will be a difference in both side effects under anti-VEGF free therapies such as durvalumab/tremelimumab.

Furthermore, we report a significantly higher ORR under atezo/bev compared with lenvatinib. However, these effects did not translate into a higher OS or PFS. In this regard, we observe a trend toward a higher OS under atezo/bev, with an HR of 0.78 and a *p* value of 0.058. In our opinion, these results could indicate that the power of the study is too low to demonstrate statistical significance and might explain why the effect on ORR did not translate into hard endpoints such as OS or PFS.

However, these data also need to be interpreted in the context of the fact that at the time of the data cut-off, there was a higher percentage of patients who received follow-up treatment in the lenvatinib group (52 *vs*. 30%). This disparity might contribute to the underestimation of the efficacy of atezo/bev compared with lenvatinib. Furthermore, a response to checkpoint inhibitor therapy may differ from the response to tyrosine kinase inhibitor therapy. There are effects reported from long-term responses, which are scarce but could make a fundamental difference in an individual situation, as long-term survival is feasible in these cases.

Therefore, the reported results on efficacy should not simply be interpreted as not having reached statistical significance; rather, they should be weighted more differentially, as discussed here.

The present study allowed us to investigate a large dataset from tertiary HCC centers in Germany and Austria. We believe that a comparison between the two first-line therapies regarding efficacy and safety is urgently needed. Given that it is unlikely that industry partners will conduct such a study, investigating patient cohorts appears to be the most appropriate available approach. A strength of our study is the examination of a pure real-world population, which is often not adequately represented in HCC trials, as these trials typically recruit patients with excellent liver function. We acknowledge a bias in our study attributable to its non-randomized and non-prespecified prospective data collection nature. Nevertheless, it is noteworthy that only a minority of patients received lenvatinib after the approval of atezo/bev. In our opinion, this observation makes the possibility of a selection bias, which could be relevant for interpreting these results, less likely. We infer from this distribution that almost all patients received atezo/bev from this point onward, rendering the two groups more accurately comparable, as both were favored in their respective times.

Therefore, we believe that our data can provide reliable evidence on the safety and efficacy of both agents, acknowledging that the nature of a retrospective study inherently harbors limitations.

One potential limitation worth noting in this study is that patients were exclusively recruited from tertiary centers. This fact may restrict the generalization of the presented results.

Taken together, we provide a large real-world comprehensive analysis of bleeding rates under atezo/bev and lenvatinib in a well-characterized cohort. Bleeding rates and thromboembolic events did not differ between atezo/bev and lenvatinib. Therefore, we believe that neither the risk of bleeding nor the risk of thromboembolic events should solely guide the selection of therapy between atezo/bev and lenvatinib.

## Financial support

This study was initiated by the IMMUreal study group and was supported by the Bavarian Cancer Research Center (BZKF). The funding bodies had no role in the design of the study, the collection, analysis, interpretation of data, or the writing of the manuscript.

## Authors’ contributions

Designed the study: FPR and NBK. Conducted data analyses: FPR and NBK. Wrote the manuscript: FPR and NBK. Were involved in the data collection and preparation of the manuscript: all co-authors.

## Data availability statement

Data are available upon request.

## Conflicts of interest

NBK has received reimbursement of meeting attendance fees and travel expenses from EISAI and lecture honoraria from the Falk Foundation and AstraZeneca. UE has received honoraria for lectures from AstraZeneca, the Falk Foundation, Ipsen, and Novartis and travel support from AstraZeneca and Biotest. She has served as an advisory board or steering committee member to AstraZeneca, Bayer, EISAI, and MSD. KB has received honoraria for lectures from Ipsen. MP served as a speaker and/or consultant and/or advisory board member for AstraZeneca, Bayer, Bristol-Myers Squibb, Eisai, Ipsen, Lilly, MSD, and Roche and received travel support from Bayer, Bristol-Myers Squibb, Ipsen, and Roche. BS received grant support from AstraZeneca and Eisai; speaker honoraria from Eisai; and travel support from AbbVie, AstraZeneca, Ipsen and Gilead. OÖ received honorarium from Bayer. MV has received honoraria for her speaker, consultancy, and advisory roles from Amgen, AstraZeneca, Bayer, BMS, EISAI, Ipsen, Lilly, Merck Serono, MSD, Nordic Pharma, Roche, Servier, and Sirtex. SJG has received travel expenses from Gilead and Ipsen. FF has received honoraria for lectures from AstraZeneca, MSD, Pfizer, and Roche and reimbursement of meeting attendance fees and travel expenses from Merck KGaA and Servier. He has served as an advisory board or steering committee member to AstraZeneca, BMS, Eisai, and Roche. ENDT has served as a paid consultant for AstraZeneca, Bayer, BMS, EISAI, Eli Lilly & Co, Pfizer, Ipsen, and Roche. He has received reimbursement of meeting attendance fees and travel expenses from Arqule, AstraZeneca, BMS, Bayer, Celsion, and Roche and lecture honoraria from BMS and Falk Foundation. He has received third-party funding for scientific research from Arqule, AstraZeneca, BMS, Bayer, Eli Lilly, and Roche. AG is an advisory board or steering committee member to AbbVie, Alexion, Bayer, BMS, CSL Behring, Eisai, Falk, Gilead, Heel, Intercept, Ipsen, Merz, MSD, Novartis, Pfizer, Roche, Sanofi-Aventis, and Sequana and a speaker for Advanz. FPR has received honoraria for lectures, consulting activities, and travel support from the Falk Foundation, AbbVie, Gilead, Ipsen, AstraZeneca, Roche and Novartis. All other authors report no conflicts of interest. MM, LSJ, CL, LB, AW, HBL, VZ, LY, JS, IP, MR, FS, and JM have nothing to declare.

Please refer to the accompanying ICMJE disclosure forms for further details.
