# Supplementary material for: Atezolizumab/bevacizumab or lenvatinib in hepatocellular carcinoma: Multicenter real-world study with focus on bleeding and thromboembolic events
Source: JHEP Rep. 2024 Apr 8;6(6):101065. doi: 10.1016/j.jhepr.2024.101065 (PMC11126929; doi:10.1016/j.jhepr.2024.101065)
Supplement: Multimedia component 1 [file mmc1.pdf]

Atezolizumab/bevacizumab or lenvatinib in hepatocellular carcinoma: Multi-center real world study with focus on bleeding and thromboembolic events

Najib Ben Khaled, Marie Möller, Leonie S. Jochheim, Catherine Leyh, Ursula Ehmer, Katrin Böttcher, Matthias Pinter, Lorenz Balcar, Bernhard Scheiner, Alexander Weich, Hans Benno Leicht, Valentina Zarka, Liangtao Ye, Julia Schneider, Ignazio Piseddu, Osman Öcal, Monika Rau, Friedrich Sinner, Marino Venerito, Simon Johannes Gairing, Friedrich Förster, Julia Mayerle, Enrico N. De Toni, Andreas Geier, Florian P. Reiter

Table of contents

Table S1……………………………………………………………………………………2
Table S2……………………………………………………………………………………4
Fig. S1……………………………………………………………………………………...4
Fig. S2……………………………………………………………………………………...5

Risk factors gastrointestinal bleeding in cirrhotic patients treated with atezobev						
**Variable**	**Univariate Odd's ratio**	**95% (CI)**	**p-value**	**Multivariate Odd's ratio**	**95% (CI)**	**p-value**
**Age**	**0.96**	**0.93 to 1.0**	**0.04**	0.96	0.93 to 1.0	0.05 ns
Sex, male	0.94	0.42 to 2.4	0.89			
CPS B-C (CPS A as reference)	1.4	0.61 to 2.9	0.42			
MVI	0.77	0.32 to 1.7	0.54			
Platelets	1.0	0.99 to 1.0	0.17			
Spleen size	1.1	0.99 to 1.3	0.09			
Presence of varices	1.7	0.78 to 3.7	0.19			
**History of variceal bleeding**	**3.3**	**1.2 to 8.9**	**0.02**	**3.1**	**1.1 to 8.3**	**0.03**
NSBB	0.56	0.25 to 1.2	0.14			
Prior EBL	1.8	0.77 to 4.0	0.15			
Anticoagulation	1.4	0.69 to 2.8	0.34			
Antiplatelets	0.76	0.31 to 1.7	0.53			

Risk factors variceal bleeding in cirrhotic patients treated with atezobev			
**Variable**	**Univariate Odd's ratio**	**95% (CI)**	**p-value**
Age	0.95	0.91 to 1.0	0.06
Sex, male	1.8	0.47 to 12	0.46
CPS B-C	2.1	0.69 to 6.0	0.17
MVI	1.3	0.41 to 4.2	0.61
Platelets	1.0	0.99 to 1.0	0.69
Spleen size	1.1	0.93 to 1.4	0.24
Presence of varices	3.1	0.92 to 14	0.09
Esophageal varices grade I	2.0	0.42 to 10	0.38

**Esophageal varices grade II-III**	**5.4**	**1.3 to 26**	**0.02**			
History of variceal bleeding	1.7	0.25 to 6.7	0.51			
NSBB	0.79	0.24 to 2.3	0.68			
Prior EBL	1.6	0.43 to 5.0	0.42			
Anticoagulation	0.65	0.23 to 1.9	0.41			

**Risk factors non-gastrointestinal bleeding in cirrhotic patients treated with atezobev**						
**Variable**	**Univariate Odd's ratio**	**95% (CI)**	**p-value**	**Multivariate Odd's ratio**	**95% (CI)**	**p-value**
Age	1.0	0.97 to 1.1	0.71			
Sex, male	0.68	0.19 to 1.9	0.50			
CPS B-C (CPS A as reference)	0.24	0.037 to 0.83	0.05 ns	0.23	0.036 to 0.82	0.05 ns
Platelets	1.0	1.0 to 1.0	0.83			
**Anticoagulation**	**2.3**	**1.0 to 5.2**	**0.05**	**2.3**	**1.0 to 5.4**	**0.04**
Antiplatelets	0.87	0.31 to 2.2	0.77			

Table S1 Potential risk factors for gastrointestinal bleeding, variceal bleeding and non-gastrointestinal bleeding in patients with liver cirrhosis treated with atezo/bev. Odd’s ratios for risk of bleeding were calculated using a univariate followed by a multivariate logistic regression model. CI: confidence interval, CPS: Child-Pugh score, EBL: endoscopic band ligation, MVI: macrovascular invasion, ns: non significant, NSBB: non-selective beta-blockers.

Event	Atezolizumab + bevacizumab n=278	Lenvatinib n=125	OR (95% CI)	P-value
Bleeding, n (%)	44 (15.8)	14 (11.2)	1.49 (0.80-2.78)	0.28
Variceal hemorrhage, n (%)	9 (3.2)	4 (3.2)	1.01 (0.31-3.0)	>0.99
Thromboembolic events, n (%)	18 (6.5)	5 (4.0)	1.66 (0.62-4.20)	0.36

Table S2. Bleeding and thromboembolic events in the first 3 months of therapy, excluding patients who experienced early mortality as defined by death occurring in the first 3 months. Fisher's exact test was used for comparing categorical variables.

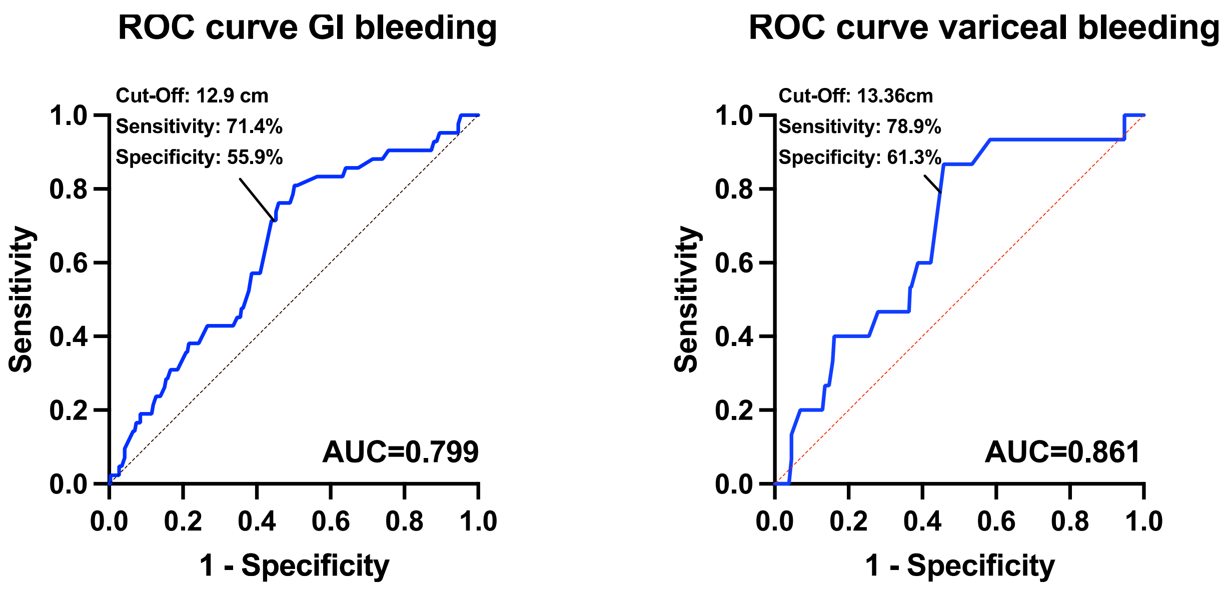

Fig. S1. The receiver-operating characteristics (ROC) curve showing the sensitivity and specificity of baseline spleen size cut off values to predict A: GI bleeding and B: variceal bleeding. Abbreviations: AUC, area under the curve.

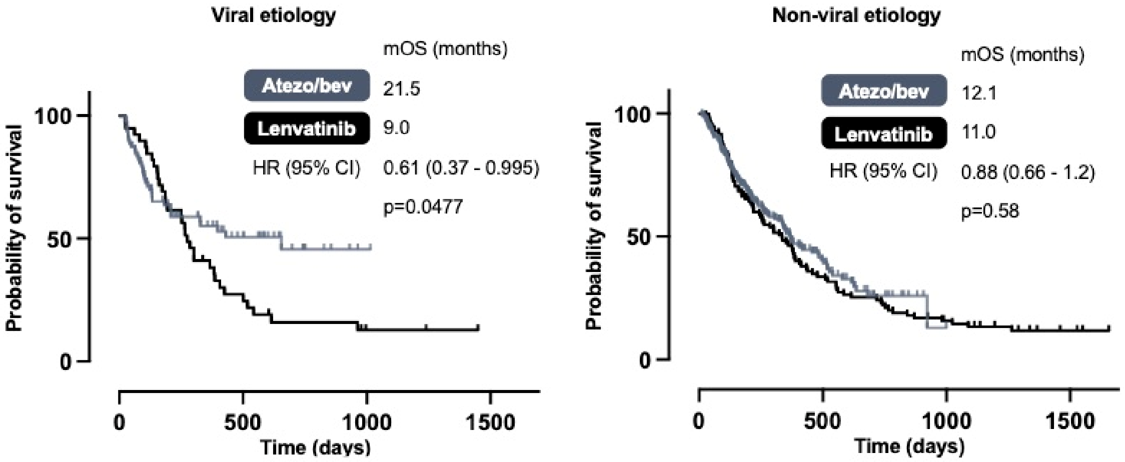

Fig. S2. Efficacy of therapy in patients who received Atezo/bev or Lenvatinib as systemic first-line therapy with respect to viral and non-viral etiology. Kaplan-Meier analysis was performed to calculate the median survival, survival was compared using the log-rank test. Abbreviations: CI, confidence interval; HR, hazard ratio; mOS, median overall survival.
