# Supplementary material for: Atezolizumab/bevacizumab or lenvatinib in hepatocellular carcinoma: Multicenter real-world study with focus on bleeding and thromboembolic events
Source: JHEP Rep. 2024 Apr 8;6(6):101065. doi: 10.1016/j.jhepr.2024.101065 (PMC11126929; doi:10.1016/j.jhepr.2024.101065)
Supplement: Multimedia component 2 [file mmc2.pdf]

JOURNAL OF HEPATOLOGY

Journal of Hepatology

CTAT methods

Tables for a "Complete, Transparent, Accurate and Timely account" (CTAT) are now mandatory for all revised submissions. The aim is to enhance the reproducibility of methods.

- Only include the parts relevant to your study
- Refer to the CTAT in the main text as 'Supplementary CTAT Table'
- Do not add subheadings
- Add as many rows as needed to include all information
- Only include one item per row

If the CTAT form is not relevant to your study, please outline the reasons why:

Only section 1.7 applies as the submitted work is not based on laboratory experiments but clinical data.

1.1 Antibodies

Name	Citation	Supplier	Cat no.	Clone no.

1.2 Cell lines

Name	Citation	Supplier	Cat no.	Passage no.	Authentication test method

1.3 Organisms

Name	Citation	Supplier	Strain	Sex	Age	Overall n number

1.4 Sequence based reagents

Name	Sequence	Supplier

1.5 Biological samples

Description	Source	Identifier

1.6 Deposited data

Name of repository	Identifier	Link

1.7 Software

Software name	Manufacturer	Version
GraphPad Prism 9	GraphPad Software; San Diego, CA, USA	**9**

1.8 Other (e.g. drugs, proteins, vectors etc.)

1.9 Please provide the details of the corresponding methods author for the manuscript:

Priv.-Doz. Dr. Florian Reiter

Division of Hepatology, Department of Medicine II

University Hospital Würzburg

Würzburg, Germany

Phone: +49 931 201 40811; Fax: +49 931 201 640023

Email: Reiter_F@ukw.de

2.0 Please confirm for randomised controlled trials all versions of the clinical protocol are included in the submission. These will be published online as supplementary information.
